# Submuscular plating of the femur through an anterior approach after bone distraction

**DOI:** 10.1007/s11751-016-0274-2

**Published:** 2017-01-07

**Authors:** Federico Persico, Gabriel Fletscher, Mauricio Zuluaga

**Affiliations:** Osteoarticular Diseases Institute, Imbanaco Medical Center, Cali, Colombia

**Keywords:** Conversion, Bone distraction, Anterior approach, MIPO technique

## Abstract

The method of osteogenesis by distraction is a known technique in orthopaedics for the management of bone defects secondary to trauma, infections or tumours. New strategies have been developed for decreasing the external fixator time. The use of the minimally invasive plate osteosynthesis technique is a secure approach through a percutaneous fixation technique in the anterior aspect of the femur that permits minimal dissection of the soft tissues while preventing cross-contamination with the pin tracts of the external fixators. The goal of this article is to show that a new surgical technique, to preserve the benefits related to the internal fixation and at the same time decrease the risk of infection, can be used to perform femoral plating after bone distraction with a low contact plate through an anterior approach to the femur while still taking adequate care of the soft tissues.

## Introduction

Distraction osteogenesis, initially described by Ilizarov, is established for the management of bone defects after trauma, infections or tumours [1, 2]. Complications associated with the prolonged use of the external fixators are high [3], especially for the pin tract infections. This ranges from 7.5 to 80% [4] with a potential failure of the fixator for 30% [5], and an 8–30% risk of re-fracture and deformity after the removal of the external fixator [6].

New strategies have been developed for decreasing the external fixator time and to reduce complications such as infection, joint stiffness, deformity and re-fractures in the regenerated segment. Several authors have published their experience using different internal fixation methods after the distraction period; these include techniques such as lengthening over locking plates or nails [7], nail insertion or plating after the lengthening [8, 9] with the fixator in situ, or removal of the external fixator prior to the application of the plates in one [10] or two surgical stages [11, 12].

If inserting plate fixation after external fixation, the surgical approach should avoid previous pin sites to decrease the risk of cross-contamination and infection. Use of the MIPO technique through the anterior aspect of the femur permits minimal dissection of the soft tissues while preventing cross-contamination with the pin sites of the external fixator. This report describes this surgical technique, allowing the benefits of internal fixation with a lower risk of infection. A locking angle stable plate is used as there is evidence to suggest these plates are useful as internal fixators [13] providing stability while resisting bending.

## Indications

The introduction of combination techniques of internal and external fixation during bone lengthening has made it possible to remove the frame after the end of the distraction period and substitute an alternative form of stabilization. All patients, regardless of the maturity found in the regenerated bone after the distraction period, are amenable to plate substitution; where the maturity of the bone regenerate is in question, or in situations in which a presumption of poor prognosis of consolidation at the docking site level is of poor quality, the procedure is performed with bone grafting.

### Radiological assessment

The anteroposterior (AP) and lateral radiograph of the femur are important for the features in the bone distraction performed: there needs to be an evaluation of the position of the regenerate column; the segments of femur proximal and distal to it for anchorage of plate screws and an overall estimation of plate length to ensure mechanical stability. It is also important to review the position of the external fixation pins in each of the bony segments as some may need removal at the beginning of the surgical intervention.

### Antibiotic prophylaxis

In this technique where internal fixation is introduced through surgical wounds away from the pin sites of external fixation, it is not necessary to use an antibiotic scheme prior to the surgical procedure other than the dose for routine prophylaxis as is used for all other surgical intervention. We use a first-generation cephalosporin.

## Surgical technique

### Patient position

**Fig. 1:**
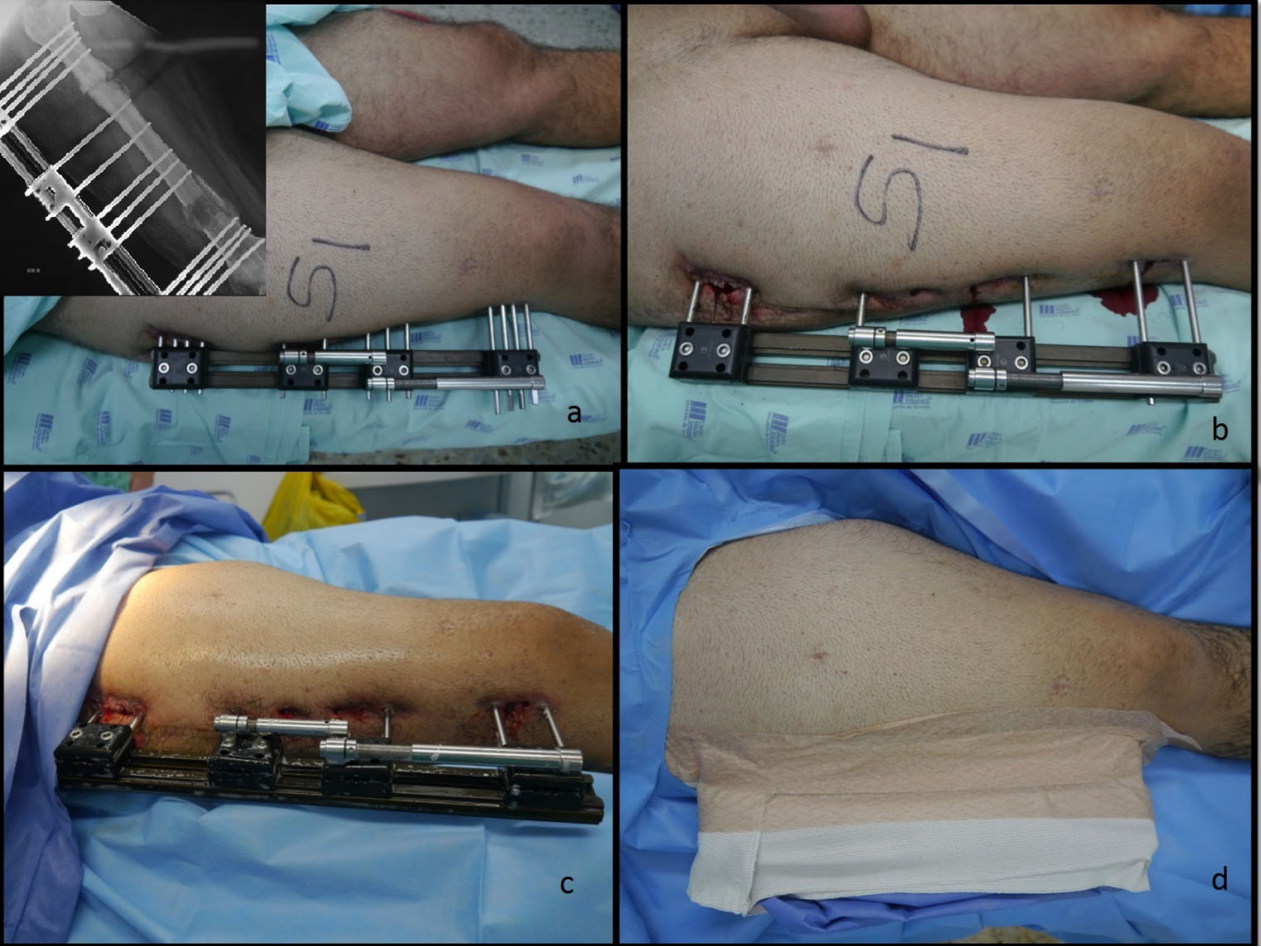
Patient position and draping. **a** Preoperative frame appearance. **b** and **c** Removal of half pins without jeopardizing the stability of the regenerated bone. **d** The frame is isolated from the surgical field after prepping and draping to avoid cross-contamination

The patient is in a supine position on the surgical table. A raise or cushion is placed under the buttock region to ensure good access for limb preparation and to avoid any contamination to the external fixator from the surgical table. The limb elevation should not limit rotation of the extremity such that fluoroscopic evaluation of the femur in the AP projection is unimpeded (Fig. 1a).

As there is a risk of infection with this manner of conversion to plating after distraction osteogenesis by external fixation, the surgical management of the soft tissues and of the operative field is of vital importance to prevent bacterial contamination within the pin-free zone. The technique is divided into *three phases*.

#### Phase 1: partial removal of the external fixator and isolation of the external fixator from the surgical field

The intermediate pins within a single pin clamp are removed, distal and proximal to the regenerate segment, taking care to not affect the stability of the external fixation (Fig. 1b, c). The remaining parts of the external fixator are then wrapped using sterile surgical towels to exclude it from the sterile operative field (Fig. 1d).

#### Phase 2: MIPO technique

**Fig. 2:**
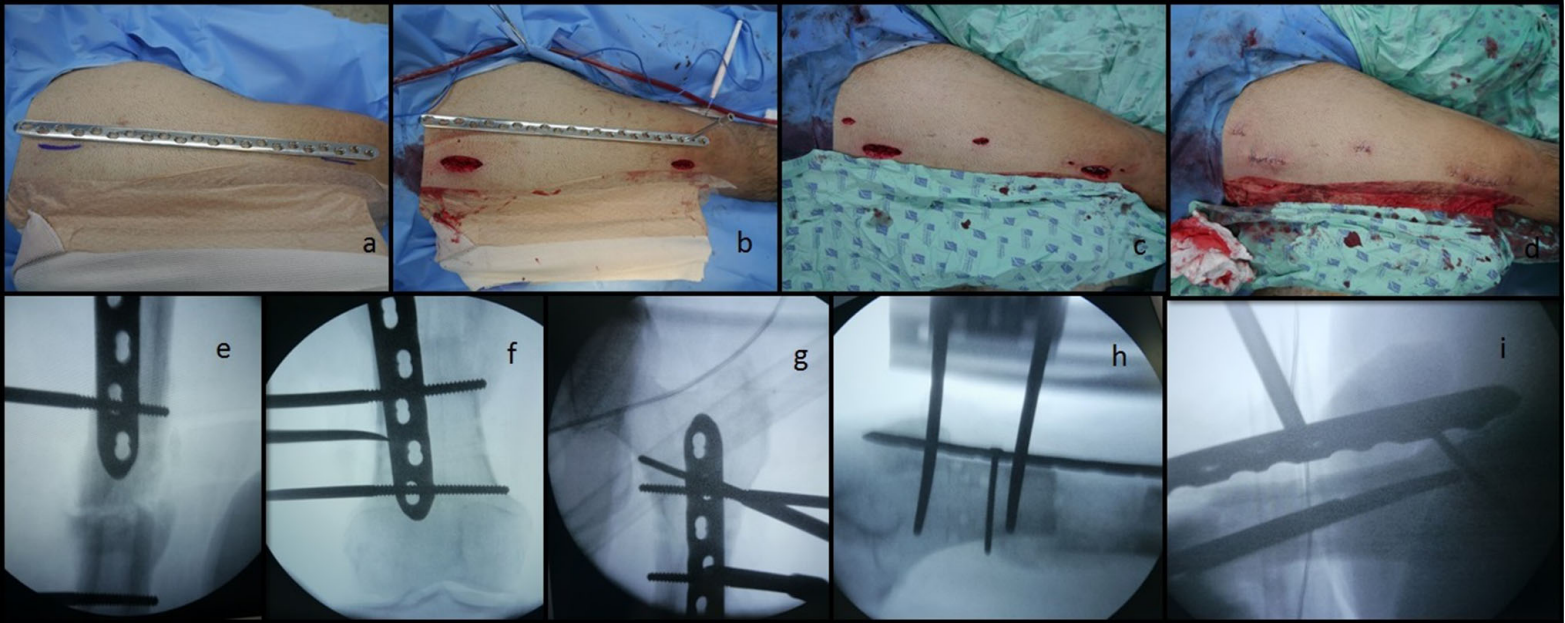
Anterior MIPO technique **a** under image intensifier the correct plate size is chosen. **b**–**c**–**d** Skin incisions. **e** Plate at the docking site. **f**–**g** Under intensifier check the position of the plate over the *centre* of the bone. **h**–**i**: Start the fixation with cortical screws in the proximal and distal segment

The length of the regenerated bone and the proximal and distal bone segments allow selection of the appropriate plate length; the best fit is confirmed via fluoroscopy (Fig. 2a). Most often LCP 4.5-mm plates, between 18 and 24 holes, are used in adults, so preoperative planning is necessary to ensure the availability of long plates.

The minimally invasive anterior approach combines exposure of proximal and distal femur through two incisions of approximately 2 inches long (Fig. 2b). In the proximal portion, the incision is made in the anterior thigh around the level of the subtrochanteric region. After dissection of the subcutaneous tissue, the interval between tensor fasciae lata and the anterior rectus femoris muscles is developed to reach the metaphyseal portion of the femur. Some splitting of the underlying vasti muscles is needed to reach the anterior surface of the femur. In the distal end of the incision, blunt dissection pushes the descending branch of the lateral femoral circumflex artery and some lateral branches of the femoral nerve out of the way.

The suprapatellar approach is performed in the distal region by making a longitudinal incision of 2 inches (5 cm) along a line that connects the lateral edge of the patella to the anterior superior iliac spine. The femoral surface is reached by sharply dividing the interval between the lateral edge of the quadriceps tendon and vastus lateralis. Beneath this layer, the vastus intermedius is split longitudinally, exposing the distal femoral metaphysis.

The two incisions are then connected in the supraperiosteal and submuscular plane by blunt dissection in a retrograde and anterograde fashion.

### Plate positioning and fixation

The selected LCP is introduced in the submuscular plane parallel to the femur diaphysis. During this step, it is helpful for fluoroscopic monitoring in order to determine areas in which the plate does not move forward with ease or impinges, a situation that is associated usually with the presence of prominent bone callus or irregularities in the regenerated bone (Fig. 2c). In addition, when dealing with immature regenerate bone segments, caution is advised when sliding the plate submuscularly in the event the plate penetrates the soft regenerate instead of lying alongside it.

After the placement of the plate on the femoral shaft, it is recommended to check the centring of the plate in the femoral diaphysis prior to the start screw insertion and fixation (Fig. 2d). Fixation of the plate starts with the placement of one cortical screw each to the proximal and distal segment first and then completed with at least three additional locking screws to each end of the plate (Fig. 2e).

Bone grafts are inserted into the regenerate bone segment or at the docking site (in bone transport) if considered relevant during the preoperative planning. A layered wound closure includes repair of the extensor mechanism distally. The wounds are then sealed with sterile dressings prior to removal of the remaining components of the external fixator.

#### Phase 3 : removal of the external fixator and debridement of external fixator pin sites

**Fig. 3:**
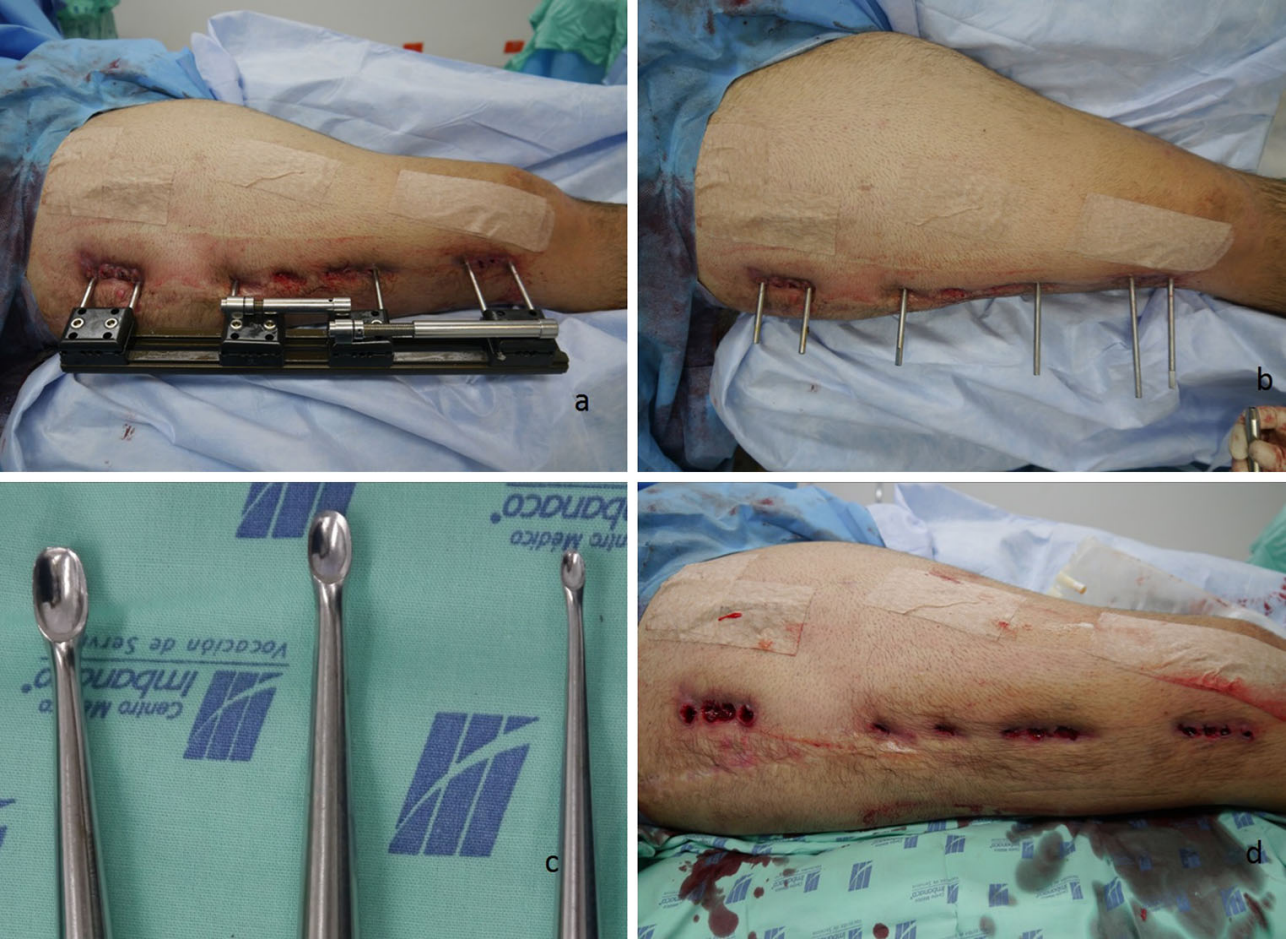
Frame removal. **a** Isolation of surgical wounds. **b** Removal of the frame. **c** Three different sized curets to perform the debridement of the pin tracts. **d** Appearance of the pin tract after the debridement

For debridement of the soft tissues and bone curettage of the femur, three curettes of different sizes are used in order to perform a debridement of the granulomatous reactions around the pin sites (Fig. 3a–c). With the larger curette, a superficial debridement is performed; the intermediate-sized curette then removes the soft tissue more deeply and finally the smaller-sized curette is then passed to within the bony track to curette from within to out (Fig. 3d). At the end of the procedure, the pin sites are covered with sterile dressings.

### Postoperative care

In the postoperative period, partial weight bearing is advised until maturation of the bone regenerate is complete. The first-generation oral cephalosporin is used during the 1st week after surgery. Stitches are removed from the wounds at 2 weeks. No additional care is required for pin sites as these heal quickly by secondary intention.

## Cases series

From January 2012 to January 2014, 30 patients underwent femoral lengthening followed by plating through the described approach. The patients included 17 men and 13 women with an average age of 51.5 years (range between 20 and 83). The spectrum of pathologies treated was 66.6% septic non-union of the femur; 16.6% aseptic non-union; 13.3% fractures; and 3.3% (in one case) for a bone tumour. For all cases, successful healing was recorded.

 All patients started lengthening at a distraction of 1 mm/day and in some cases adjusted later to 0.75 mm/day according to the findings during radiological monitoring. The average time of bone distraction was 74 days (range 52–96 days).The average size of the regenerate column was 6.3 cm (range between 4.6 and 8 cm). The external fixation index (EFI) was 0.4 month/cm (range 0.37–0.44 month/cm). The average total treatment time, calculated from the time of the placement of the external fixator until union was achieved, was 248 days (range between 220 and 276 days). In 10 patients, the procedure was combined with demineralized bone matrix (DePuy Synthes), 6 in the docking site and 4 in the regenerated bone.

There were no complications during the surgical procedure. In the postoperative follow-up, there was one documented case of superficial infection which improved with oral antibiotics but did not require removal of the implant. In the cases involving septic non-unions, there were no recurrences of previous osteomyelitis. One patient presented with failure of the distal plate fixation; this was considered to be secondary to the poor bone quality and insufficient hold on the short distal segment of the femur with three locking screws. Prior to the surgical procedure of substituting the plate for the external fixator, 17 of the 30 patients showed an extension contracture of the knee (loss of flexion range). At one year postsurgical follow-up, 12 had a knee range of mobility that was equal to or greater than 70°. There were no fractures or angular deformities in the evaluated patients.

## Discussion

**Fig. 4:**
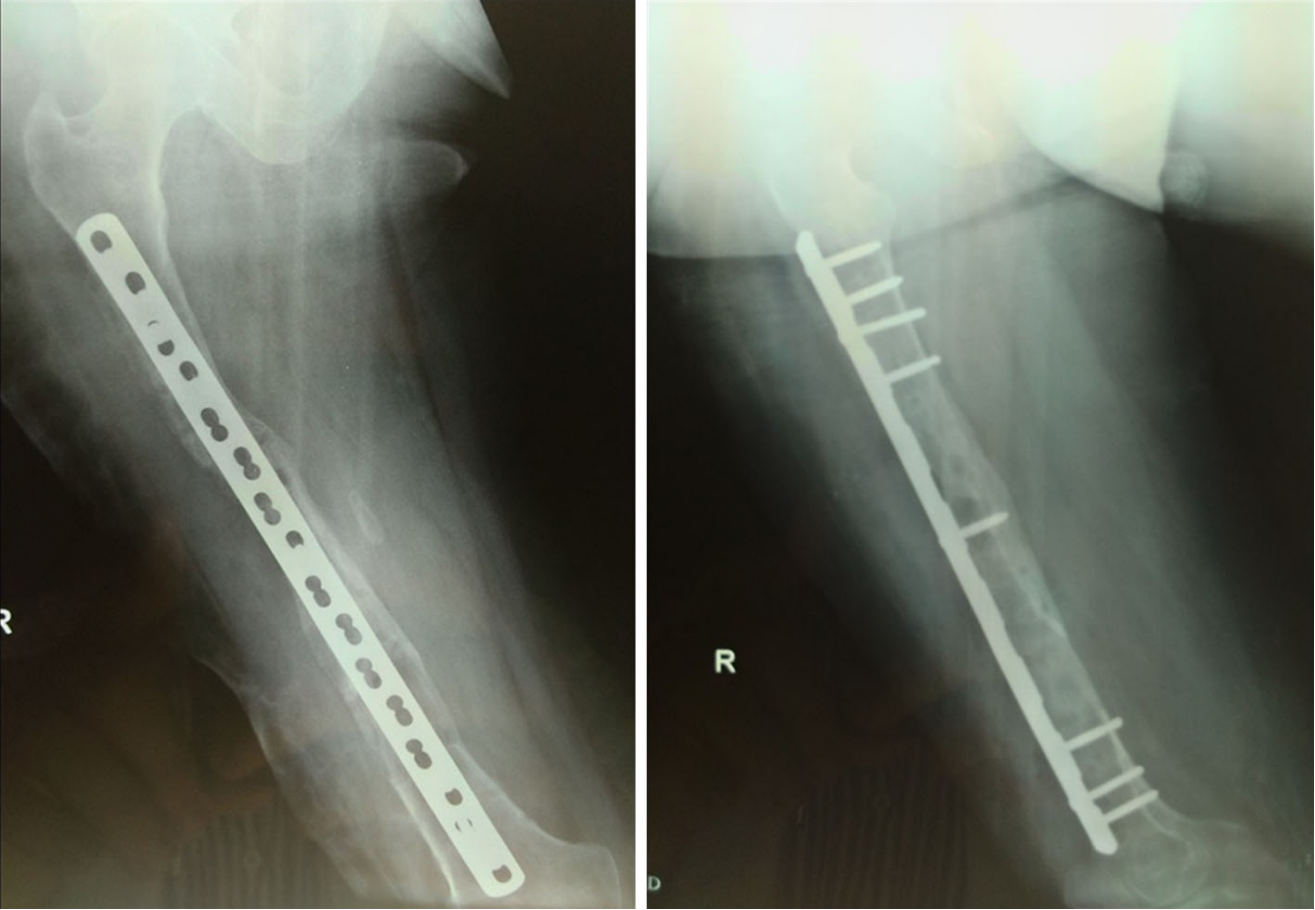
Follow-up after 2-month index procedure. Note the mature bone healing at the docking site with good alignment and fixation

Plating after femoral lengthening is offered to patients treated with external fixators for bone lengthening or bone transport. The use of locking plates has the same advantages of the intramedullary nails but without risks of intramedullary infections, growth plate damage, avascular necrosis or fat embolism. The technique is applicable at any age and in any bone. The locking plate design averts loosening and protects the bone regenerate from deformities or bending after removal of the frame (Fig. 4) [14, 15]. With the described technique, there are two principal advantages: (1) use of a pin-free zone for decreasing the risk of infection and (2) a single surgical procedure without prior treatment with antibiotic.

Different techniques are used for managing bone defects amongst which that most used are distraction osteogenesis, free transfer of vascularized grafts and, more recently, the induced membrane technique before bone grafting [16]. Distraction osteogenesis is dependent on the use of external fixation mainly. Complications associated with the prolonged use of the external fixation and the inconvenience caused by the external fixator have decreased the popularity of this method for the patients. It was suggested that removal of the frame as soon as the distraction period is completed will improve treatment compliance but alternative methods of stabilization had to be used when external fixation is removed before regenerate consolidation is complete. Given the potential risk of infection from contamination found in the medullary canal with the use of intramedullary nails after external fixation, plate fixation has become a good alternative for stabilization, but the current evidence does not provide conclusive recommendations of one method over the other. With the development of the locking plates, a stable fixed angle design that works as an internal fixator was created. This can prevent deformities when used as a bridge across a regenerated bone segment. In addition, these plates can be inserted percutaneously thereby decreasing soft tissue injury and leaving a better cosmetic result. This technique avoids risk of intramedullary infections during the lengthening over intramedullary nails [10]. It prevents damage to the endosteal circulation from reaming and is without risk of damage to the physis or from avascular necrosis of the femoral head when the nail is inserted through the piriformis fossa in a paediatric patient [12].

Several authors have reported their results using the MIPO and LCP techniques to decrease the external fixation time [17, 18]. In 2006, Iobst and Dahl presented a series of 6 cases in children (mean age 7.6 years) with 5 femurs and 1 tibia using the Ilizarov frame; lengthening was carried out over a locked plate fixed proximally (average 3.5-cm lengthening). The external fixation index (EFI) was 0.42 month/cm. At the end of the 10-month follow-up, 3 patients had a procurvatum deformity with one requiring surgical correction. Other reported complications included 1 premature consolidation of the regenerate, 1 fracture above the plate 3 months after osteosynthesis surgery, 1 pin track infection and 1 joint contracture. Based on their findings, the authors noted the decreased time with external fixation allowed recovery of joint mobility faster, while avoiding contractures and subluxations [15].

This case series did not encounter serious complications. There was a considerable decrease in the external fixation time when plate fixation was used as soon as distraction was completed. A superficial infection of the one surgical wound was documented but improved with oral antibiotics. The loss of the mobility of the knee, usually when in external fixation, improved significantly to a functional range when substitution by plate stabilization was carried out and further physiotherapy implemented.

## Conclusion

Early substitution to plate internal fixation after lengthening by external fixation is a method that facilitates patient recovery. The anterior approach combined with MIPO enables surgery to avoid the previous external fixation pin sites. This technique allows the combination of internal and external fixation to be carried out with a lower risk of deep infection.
